# Improving Sleep Quality and Well-Being in Institutionalized Older Adults: The Potential of NESA Non-Invasive Neuromodulation Treatment

**DOI:** 10.3390/geriatrics10010004

**Published:** 2025-01-03

**Authors:** Aníbal Báez-Suárez, Virginia Báez-Suárez, Laissa Saldanha, Martín Vílchez-Barrera, Andrea Hernández-Pérez, Raquel Medina-Ramírez

**Affiliations:** 1 Faculty of Health Sciences, University of Las Palmas de Gran Canaria, 35016 Las Palmas, Spain; saldanhalaissa@gmail.com (L.S.); martin.vilchez@ulpgc.es (M.V.-B.); raquel.medina@pdi.atlanticomedio.es (R.M.-R.); 2 Centro Sociosanitario Queen Victoria, 35011 Las Palmas, Spain; v.baez@queenvictoria.es; 3 Faculty of Health Sciences, University of La Laguna, 38071 Santa Cruz de Tenerife, Spain; alu0101774411@ull.edu.es; 4 Faculty of Health Sciences, University of Atlántico Medio, 35017 Las Palmas, Spain

**Keywords:** sleep quality, geriatric nursing, autonomic nervous system, mental health, geriatrics

## Abstract

**Background/Objectives**: Ageing is associated with several cognitive, physical, and emotional changes, including a decrease in sleep quality and mental health issues. This study studies NESA (Spanish acronym for Neuromodulación Superficial Aplicada) non-invasive neuromodulation using microcurrents as something that may provide a potential improvement in the quality of sleep and general health of older adults and residents in a healthcare institution. **Methods**: This observational study recruited 24 people who were residents at a long-term care facility. Participants were divided into two groups: one intervention group, who underwent NESA therapy twice a week for a total of 20 sessions, and a control group, who did not receive this treatment. The outcomes measured include sleep quality (Pittsburgh Sleep Quality Index), diary of sleep, symptoms of depressed mood (Yesavage Geriatric Depression Scale), and quality of life with the World Health Organization Quality of Life-Old (WHOQOL-OLD). Scores were collected at baseline, after 10 and 20 sessions, and 3 months after finishing the treatment. **Results**: The intervention group exhibited a notable improvement in sleep quality (*p* = 0.05). Additionally, there were fewer nocturnal awakenings. The quality of life also showed better scores, especially in relation to social relations and physical and mental health, which matches the slight decrease in scores and clinical improvement regarding depressive symptoms. In contrast, the control group demonstrated no improvement in symptoms, and in some cases, there was a worsening of symptoms. **Conclusions**: Our findings indicate that NESA non-invasive neuromodulation therapy is likely to enhance sleep quality and health-related measures in institutionalized older adults. Despite the limitations of the current study, the results support the potential of NESA microcurrents to enhance the well-being of this population.

## 1. Introduction

Ageing is a complex biological process that causes physical, cognitive, and emotional changes over time [1]. With advancing age, transformations in various body systems, including the cardiovascular system, increase susceptibility to chronic conditions [2]. In the central nervous system, ageing affects cognitive and emotional functions, impairing episodic memory, cognitive processing, and the acquisition of new information, which compromises autonomy and quality of life at advanced ages [3].

In addition, ageing is associated with changes in circadian rhythms, which frequently result in sleep disorders among the elderly [4,5]. These alterations in the sleep-wake cycle, combined with increased susceptibility to mood disorders, add to the complexity of mental health challenges in this population [6]. Studies indicate that sleep fragmentation and circadian dysregulation are closely linked to heightened depressive and anxiety symptoms in older adults, suggesting a strong relationship between sleep quality and mental well-being in later life [7]. It should also be noted that sleep fragmentation has been identified as a potential early biomarker in the progression of dementia [8].

In view of this, evidence suggests that promoting slow waves during non-rapid eye movement (NREM) sleep in the elderly may have a neuroprotective effect, aiding in the elimination of β-amyloid (Aβ) and consequently reducing the risk of developing neurodegenerative diseases [9]. Furthermore, the intensification of slow waves in NREM sleep can restore proteins damaged by oxidative stress, as observed in animal models [10], suggesting that enhancing sleep quality may slow cognitive decline.

In this context, recent studies indicate that non-invasive neuromodulation targeting the regulation of the autonomic nervous system represents a promising approach to enhance sleep quality in the elderly population [11]. In addition, the results of a systematic review suggest that these interventions can improve specific aspects of sleep, such as efficiency and latency [12]. By adjusting brain activity patterns related to the sleep-wake cycle, this type of treatment has shown potential to improve well-being and, in a broad sense, quality of life in diverse populations.

However, future research is essential to optimize application protocols and clarify the neurobiological mechanisms underlying these effects, enabling increasingly effective interventions for this type of therapy [13].

The NESA microcurrents (an acronym in Spanish for Neuromodulación Superficial Aplicada) represent a non-invasive neuromodulation technique that has shown promising results in enhancing sleep quality. This approach has been effective in diverse populations, including individuals with multiple sclerosis, children with neurodevelopmental disorders, and elite athletes [11,12,13,14,15]. Through the modulation of the Autonomic Nervous System (ANS) and the neuromodulation of pathways [16], NESA microcurrents offer a way to enhance sleep and manage stress without the risks associated with pharmacological treatments.

In summary, research into the interrelationship between ageing, sleep, and non-invasive neuromodulation highlights specific interventions with the potential to promote significant advances in the health and well-being of the elderly population. In this context, the present study conducted an observational analysis aiming to investigate and quantify the impact of NESA microcurrents as an effective tool for improving health-related quality of life. Additionally, it sought to assess sleep quality and analyze potential changes in depression levels among elderly residents of a social and healthcare center.

## 2. Materials and Methods

### 2.1. Study Design

This study was designed as an observational investigation, employing a convenience sample selected based on participant characteristics and availability. The design adhered rigorously to the Strengthening the Reporting of Observational Studies in Epidemiology (STROBE) guidelines, ensuring standardization and transparency in both the conduct and reporting of the applied methods. The research was approved by the HUGCDN Research Ethics Committee (Province of Las Palmas), with the opinion issued and registered under number CEIm de Las Palmas: 2023-081-1. In addition, the study was registered with Clinicaltrial.gov (NCT05800431). Registration date: 5 April 2023.

### 2.2. Recruitment and Evaluation Process

Participants were recruited on a voluntary basis using non-probabilistic convenience sampling. A specialist conducted screening consultations, assessing compliance with the inclusion and exclusion criteria by means of a detailed anamnesis. Eligible participants received an information sheet and, upon acceptance, signed a consent form. They then completed a questionnaire for baseline characterization and initial measurements. They were then allocated to the intervention according to the schedule established in the protocol.

### 2.3. Inclusion and Exclusion Criteria

The inclusion criteria required participants to be permanent residents of the Socio-Sanitary Centre, to have a stable medical and pharmacological condition, and not to have severe cognitive impairment, allowing for the inclusion of individuals with mild cognitive impairment. Participants with contraindications to the treatment—such as pacemakers, internal hemorrhages, skin lesions, acute febrile processes, acute thrombophlebitis, and/or a phobia of electricity—were excluded. Patients with severe cognitive impairment, which could compromise adherence to the procedures, were also excluded, as were those without a signed consent form, either by the participant or their legal guardian.

### 2.4. Population

The aim of this study was to investigate elderly individuals who are permanent residents of a private long-term care facility. A total of 24 participants were included: 17 women and 7 men, with a mean age of 82.71 (±8.34).

### 2.5. Intervention

**Figure 1:**
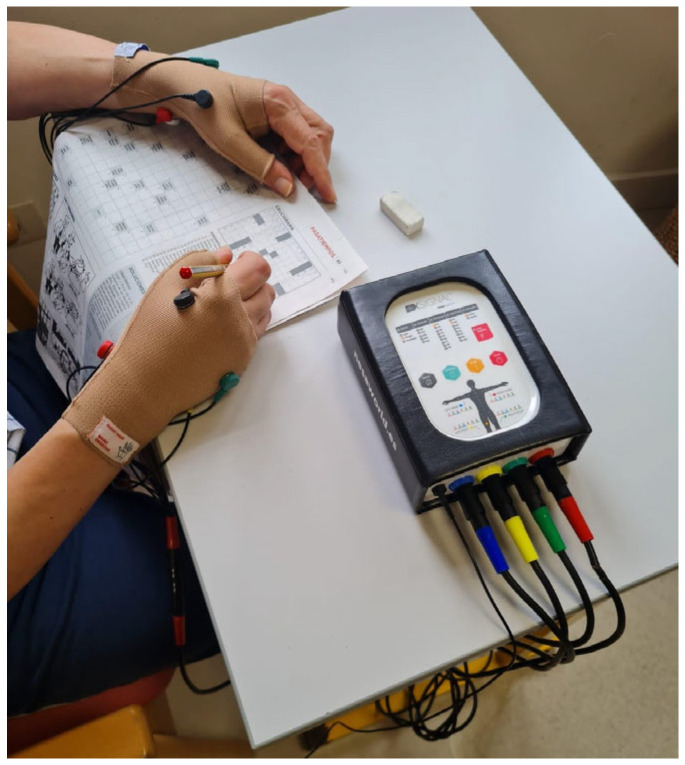
NESA microcurrent emission device and its placement on the hands using a wristband.

An attempt was made to distribute the sample into homogeneous groups. The control group maintained their usual care and treatment routine, while the intervention group received a complementary application of NESA microcurrents, carried out with a device designed specifically for this purpose, following strict guidelines. The application guidelines included sanitizing the skin with alcohol or equivalent products to ensure that oil was removed. Accessories such as gloves and socks were positioned according to standardized protocols, respecting the anatomy of the electrode application points to ensure proper adhesion (Figure 1). The targeting electrode was carefully applied to clean skin for the device to be effective. During the sessions, the presence of electronic devices close to the patient was avoided to maximize the therapeutic effects.

#### 2.5.1. Specific Guidelines of the Study

Interventions were administered twice weekly, totaling 20 sessions per participant over the treatment period. Each session lasted 60 min. Device settings were meticulously adjusted throughout the sessions to optimize participants’ physiological responses, focusing on low-intensity modulation of the autonomic nervous system to minimize potential adverse reactions and ensure procedural safety.

#### 2.5.2. Justification of Programming

The treatment schedule was structured in alignment with the study’s objectives, prioritizing institutionalized geriatric participants with mild to moderate cognitive impairment. The objectives included improving sleep quality, reducing anxiety, optimizing cognitive functions, enhancing quality of life, and monitoring comorbidities, particularly through regulation of the Autonomic Nervous System (ANS). The intervention was organized into four distinct phases:

- Phase 1—Preparation (three sessions): Program 1 employed sequential stimuli with varying frequencies (3.85 to 7.69 Hz) for gradual microcurrent introduction. Program 7 applied frequencies from 1.92 to 14.29 Hz, targeting ANS modulation and alpha wave induction. Program 8 used symmetrical stimuli at 7.69 Hz, aimed at reducing anxiety and enhancing cognitive function.
- Phase 2—Specific (three sessions): Program 1 continued at a reduced intensity for adaptation. Program 7 applied for 50% of the session, focusing on optimizing sleep and quality of life. Program 8 persisted for anxiety reduction and cognitive improvement, with targeted effects on the central nervous system.
- Phase 3—Potentiation (two sessions): Programs 7 and 8 were applied in equal proportions to intensify ANS effects and amplify therapeutic benefits.
- Phase 4—Maintenance (twelve sessions): Program 7 was applied for 45 min to achieve a cumulative effect, followed by Program 8 for 15 min at the end of each session, aimed at maintaining anxiety regulation and cognitive enhancement.

**Figure 2:**
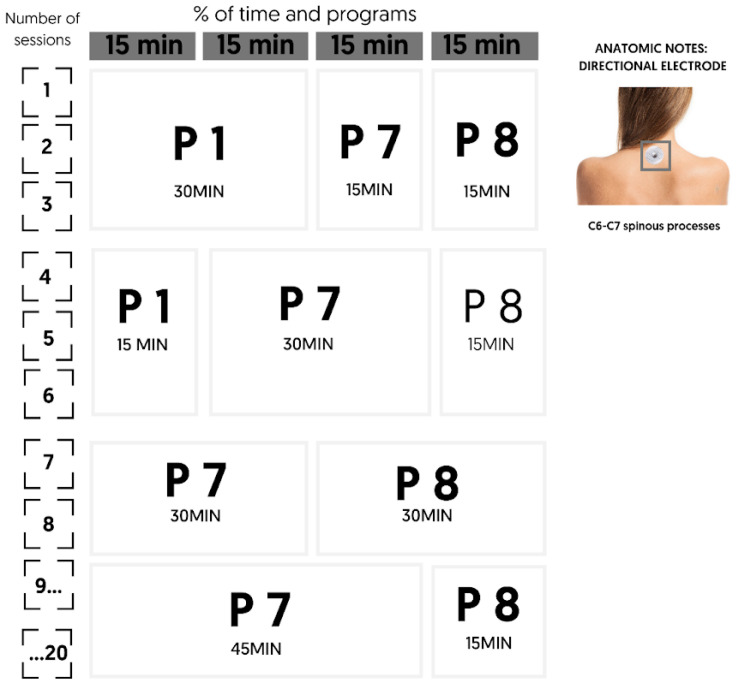
Summary of the protocol of intervention applied.

This protocol was designed to promote sustained therapeutic benefits, effectively meeting the study’s objectives while being tailored to the specific needs of the study population (Figure 2).

### 2.6. Screening and Outcome Measures

The variables analyzed included sleep quality, depressive symptoms, and quality of life. Sleep quality was monitored by the night-time monitoring team, who recorded nocturnal patterns in a standardized manner, allowing for detailed characterization throughout the intervention. Depressive symptoms were assessed using the Yesavage Geriatric Depression Scale [17,18], a validated tool with specific cut-off points for classification in elderly populations. Overall sleep quality was measured using the Pittsburgh Sleep Quality Index (PSQI), administered by the principal investigator following strict criteria [19]. Quality of life was evaluated using the Spanish version of the WHOQOL-OLD scale, developed by the World Health Organization and encompassing specific domains relevant to the elderly population [20]. A change of 0.5 to 1.0 standard deviations in the total score is generally considered clinically relevant in quality-of-life instruments; however, the specific context must be carefully considered. On the other hand, a reduction of two to three points in the global PSQI score is deemed clinically significant across various populations. These instruments were administered at four-time points: prior to treatment initiation, after 10 treatment sessions, after 20 treatment sessions, and three months after the last treatment session.

### 2.7. Data Analysis

The data collected were analyzed using Jamovi 2.3.21 to calculate descriptive and inferential statistical analysis. Firstly, descriptive statistics were performed to summarize the global characteristics of the sample. To examine intra-group differences, paired *t*-tests were used to evaluate the changes in scores over time. For group comparisons, independent sample *t*-tests were performed. All analyses were conducted at a significant level of *p* < 0.05.

## 3. Results

A total of 24 participants were included in the study, with 14 assigned to the experimental group (EG) and 10 to the control group (CG). The results of the quality of life measures (WHOQOL-OLD), sleep quality (Pittsburgh Sleep Quality Index, PSQI), number of awakenings and hours of sleep, and level of depression (Yesavage Geriatric Depression Scale) were analyzed at four-time points: at baseline (M1), after 10 sessions (M2), after 20 sessions (M3) and at three months post-treatment (M4).

### 3.1. Sleep Quality

The experimental group exhibited a notable enhancement in sleep quality following 10 sessions (M2), with a mean reduction in the PSQI total score from 11.64 (SD = 3.56) in M1 to 9.71 (SD = 4.30) in M2 (*p* = 0.05). This improvement was sustained until M4, although there was a slight regression towards the baseline level (M4: 10.43, SD = 3.32). In contrast, the control group demonstrated a progressive deterioration, particularly between M1 (10.50, SD = 3.21) and M2 (12.10, SD = 2.92) (Table 1).

### 3.2. Sleep Diary

#### 3.2.1. Sleeping Hours

No significant differences were observed between the groups or between the times within the groups. EG demonstrated a mean number of hours slept per night that remained consistent at approximately 7.48–8.43 throughout the observation period, while CG exhibited a similar pattern, with recorded values ranging from 8.63 to 8.72.

#### 3.2.2. Nocturnal Awakenings

A clinically relevant decrease in nocturnal awakenings was observed in EG, with a mean of 9.83 (SD = 7.26) in M1 and 3.50 (SD = 4.91) in M4 (*p* < 0.05 for all comparisons). Conversely, CG demonstrated a consistent level of minimal nocturnal awakenings (M1: 2.57, SD = 2.51; M4: 1.14, SD = 1.77).

### 3.3. Quality of Life

In EG, improvements were observed in all WHOQOL domains, with the greatest changes occurring after 20 sessions (M3), followed by a slight regression in M4.

- Domain 1 (Physical Health): a significant improvement was observed between M1 (50.2, SD = 18.6) and M2 (59.3, SD = 19.3), which was maintained until M3 (56.9, SD = 12.6). At M4, the values reverted to the baseline levels observed at the outset of the study.
- Domain 2 (Psychological): Significant intra-group differences were identified in EG between M1 and M3 (*p* < 0.05), with EG exhibiting a notable advantage over CG in M3 (*p* = 0.01).
- Domain 3 (Interpersonal Relationships): Significant intragroup improvements were observed in EG between M1 and M3 (*p* = 0.039). At the intergroup level, EG demonstrated superior performance compared to CG in M3, with a statistically significant difference (*p* = 0.012).
- Domain 4 (Environment): Significant alterations were observed in EG between M2 and M4 (*p* = 0.01), accompanied by intergroup discrepancies in M2 (*p* = 0.032) and M3 (*p* = 0.011).

### 3.4. Depression

The results of the analyses indicated that there were no statistically significant differences within or between the groups. Nevertheless, from a clinical perspective, there was a discernible tendency for EG to exhibit diminished depressive symptoms following the completion of 20 sessions.

## 4. Discussion

In this study, the primary aim was to investigate the impact of NESA non-invasive neuromodulation on the quality of life among elderly residents in a social and healthcare center. Previous research has demonstrated the improvement in health conditions in this population thanks to neuromodulation techniques, especially in cognitive function, physical health, and neurocognitive disorder [21,22]. Our results are consistent with these findings. Moreover, a greater improvement in specific areas can be observed, likely due to the principles of hormesis and the direct involvement of the autonomic nervous system facilitated by NESA microcurrents [23].

Regarding sleep quality, our results demonstrate a significant improvement in the intervention group, as evidenced by a reduction in test scores. The negative differences between time points indicate an enhancement in sleep quality, especially when comparing the beginning and the middle of the treatment, in which the progression stabilizes and maintains over time. As seen in other research engaging in NESA microcurrents, the improvements in sleep quality can be observed early-on in the process of the intervention and are maintained even after the end of the treatment [11,15].

As demonstrated by the results regarding health perception, a significant improvement was observed in the intervention group following the application of the treatment. Between the initial and final assessments, a substantial increase in test scores was recorded, with further improvement noted throughout the course of treatment. In contrast, the control group exhibited minimal changes or, in some cases, a slight deterioration with no clear evidence of improvement.

These findings suggest that the intervention applied to the experimental group had a positive and statistically significant impact on the perception of physical health, possibly due to the modulation of the autonomic nervous system and its influence on the biological and behavioral factors affecting the overall health of older adults [24].

In line with these results, a recent systematic review analyzed studies that utilized non-invasive neuromodulation, concluding that this approach is promising and safe for fall prevention in older adults, as it acts directly on the physiological mechanisms underlying balance and mobility [21]. This treatment is particularly relevant because it influences fundamental aspects of neuroplasticity and neurosensory integration, both of which are essential for maintaining postural stability and motor response. Additionally, the results suggest that neuromodulation may have positive effects on health perception and the fear of falling.

Another systematic review [21] reports that, during non-invasive neuromodulation treatments in older adults, there was an increase in postural stability mediated by improvements in motor control and the integration of proprioceptive stimuli in the brain. These effects were attributed to the modulation of neural activity, especially in areas responsible for sensory perception and motor coordination, resulting in significant improvements in physical perception and functionality.

The results indicate that the psychological perception of the patients in the intervention group improved consistently, particularly toward the end of the follow-up period. These enhancements were observed in a number of different fields, including positive and negative feelings, self-esteem, personal beliefs, and cognitive areas such as memory and concentration [20]. These findings indicate a favorable impact on the patient’s general mental health and resilience, which has resulted in a reduction in stress and anxiety [25]. It is well documented that other mental disorders, such as depression, are linked to lower functional abilities in elderly patients, which in turn affects their rehabilitation outcomes [26,27]. This is of particular significance when considering the growing prevalence of depression among the elderly population. The prevalence of major depressive disorder in nursing home residents is approximately twice that observed in elderly non-residents [28]. It is, therefore, imperative to consider the implications of these psychological health improvements.

In line with these findings, the experimental group showed a significant positive difference in interpersonal relationship management. This increase suggests that the neuromodulation intervention may have facilitated more effective management of the elderly participants’ social relationships, possibly through improvements in emotional regulation and stress reduction, mechanisms widely recognized in neuromodulation studies. Supporting these findings, He et al. [29] highlight that neuromodulation may have a positive impact on overall quality of life, enhancing emotional balance and improving social interaction among older adults. This impact is particularly relevant, considering that many elderly individuals face substantial challenges related to loneliness, emotional disorders, and the deterioration of social interactions, factors often associated with ageing [28,30]. In contrast, the control group showed inconsistent variations, with some deterioration observed, suggesting that the implementation of neuromodulation treatments could be an effective strategy for promoting the development of social skills in older adults.

In this study, the sleep diary was employed as the primary tool to monitor residents’ sleep hours, enabling a detailed and personalized analysis of sleep patterns. This methodology presents advantages and limitations that require careful analysis.

The use of the sleep diary in this context was justified by its practicality and feasibility in an institutional setting. In our case, nursing staff were responsible for completing the records, ensuring greater reliability in data collection due to their training and continuous access to residents. According to da Silva et al. [31], sleep diaries administered by third parties, especially in populations with cognitive impairment or functional limitations, are more reliable than self-reported ones. This is because institutionalized older adults often face difficulties in accurately completing the records due to cognitive impairments or lack of awareness of actual sleep time [32].

Furthermore, the sleep diary is considered a low-cost tool that allows the collection of detailed information on subjective sleep patterns and the factors affecting them [33]. Although electronic devices such as actigraphy or polysomnography offer greater precision, their use in geriatric institutions can be limited by cost, logistical challenges, and potential discomfort for residents. Moreover, studies like that of Natale et al. [34] have noted that combining sleep diaries with observational records, as in our case, increases the validity of the collected data.

It is important to highlight that the methodology used also has inherent limitations. Sleep diaries are subjective tools that can be influenced by observer biases, such as misperceptions or variability in interpreting sleep behaviors. Nonetheless, the prior training of nursing staff to identify specific sleep patterns sought to minimize these biases, consistent with previous recommendations in the literature [35].

This study faced several limitations that should be considered when interpreting the results. The sample size was not extensive, primarily due to the necessity of adapting to the dynamics of the institutional setting and the exclusion of certain participants. The outbreak of COVID-19 led to the isolation of some users, which not only restricted their participation but also resulted in a decline in their cognitive abilities, further reducing the eligible population. Consequently, while the findings regarding improvements in anxiety levels and the quality and quantity of sleep relative to the control group are promising, the limited sample size constrains the generalizability of these results to the broader institutionalized older adult population.

Future studies should aim to include larger and more diverse sample sizes to validate these findings and enable a broader application of the results. Additionally, incorporating complementary objective measures of sleep, such as actigraphy, alongside sleep diaries could enhance the reliability and depth of the data collected. Further research might also explore the long-term effects of non-invasive neuromodulation on sleep and anxiety in this population, as well as its potential benefits in other areas of mental and physical health.

## 5. Conclusions

In conclusion, the implementation of non-invasive neuromodulation using the NESA system demonstrates potential as an innovative approach to improving sleep and reducing anxiety in institutionalized older adults. Despite the limitations in sample size and external factors, such as the COVID-19 pandemic, the study provides relevant insights that support the feasibility and preliminary efficacy of this intervention. However, caution should be exercised in generalizing these findings, and further research is warranted to confirm and expand upon the observed benefits. This study highlights the need for tailored interventions that consider the unique challenges and opportunities present in institutionalized settings, ultimately aiming to improve the quality of life for older adults.

## Figures and Tables

**Table 1:** Mean and standard deviation per moment and group in PSQI questionnaire.

Group	M1 (Mean ± DE)	M2 (Mean ± DE)	M3 (Mean ± DE)	M4 (Mean ± DE)
EG	11.64 ± 3.56	9.71 ± 4.30	10.43 ± 2.77	10.43 ± 3.32
CG	10.50 ± 3.21	12.10 ± 2.92	11.50 ± 2.80	11.10 ± 3.07

## Data Availability

The data supporting the results will be available upon prior request to the authors.
