# Correction: Báez-Suárez et al. Improving Sleep Quality and Well-Being in Institutionalized Older Adults: The Potential of NESA Non-Invasive Neuromodulation Treatment. *Geriatrics* 2025, *10*, 4

**DOI:** 10.3390/geriatrics10040086

**Published:** 2025-06-30

**Authors:** Aníbal Báez-Suárez, Virginia Báez-Suárez, Laissa Saldanha, Martín Vílchez-Barrera, Andrea Hernández-Pérez, Raquel Medina-Ramírez

**Affiliations:** 1 Faculty of Health Sciences, University of Las Palmas de Gran Canaria, 35016 Las Palmas, Spain; saldanhalaissa@gmail.com (L.S.); martin.vilchez@ulpgc.es (M.V.-B.); raquel.medina@pdi.atlanticomedio.es (R.M.-R.); 2 Centro Sociosanitario Queen Victoria, 35011 Las Palmas, Spain; v.baez@queenvictoria.es; 3 Faculty of Health Sciences, University of La Laguna, 38071 Santa Cruz de Tenerife, Spain; alu0101774411@ull.edu.es; 4 Faculty of Health Sciences, University of Atlántico Medio, 35017 Las Palmas, Spain

In the original publication [1], there was a mistake in Table 1. Mean and standard deviation per moment and group in PSQI questionnaire. The presented data was repeated in all columns. The corrected Table 1 appears below.

The authors state that the scientific conclusions are unaffected. This correction was approved by the Academic Editor. The original publication has also been updated.

## Figures and Tables

**Table 1:** Mean and standard deviation per moment and group in PSQI questionnaire.

Group	M1 (Mean ± DE)	M2 (Mean ± DE)	M3 (Mean ± DE)	M4 (Mean ± DE)
EG	11.64 ± 3.56	9.71 ± 4.30	10.43 ± 2.77	10.43 ± 3.32
CG	10.50 ± 3.21	12.10 ± 2.92	11.50 ± 2.80	11.10 ± 3.07
